# A Small GTPase, RhoA, Inhibits Bacterial Infection Through Integrin Mediated Phagocytosis in Invertebrates

**DOI:** 10.3389/fimmu.2018.01928

**Published:** 2018-08-30

**Authors:** Ji-Dong Xu, Meng-Qi Diao, Guo-Juan Niu, Xian-Wei Wang, Xiao-Fan Zhao, Jin-Xing Wang

**Affiliations:** ^1^Shandong Provincial Key Laboratory of Animal Cells and Developmental Biology, School of Life Sciences, Shandong University, Jinan, China; ^2^State Key Laboratory of Microbial Technology, Shandong University, Jinan, China

**Keywords:** RhoA, innate immunity, integrin dependent phagocytosis, antibacterial cellular immunity, *Marsupenaeus japonicus*

## Abstract

The Ras GTPase superfamily, including more than 100 members, plays a vital role in a number of cellular processes, such as cytoskeleton recombination, gene expression, and signaling pathway regulation. Some members of the superfamily participate in innate immunity in animals. However, there have been few studies of RhoA on this aspect. In the present study, we identified a RhoA GTPase in the shrimp *Marsupenaeus japonicus* and named it *Mj*RhoA. Expression of *MjRhoA* was significantly upregulated in hemocytes and heart of shrimp challenged with *Vibrio anguillarum*. Overexpression of *MjRhoA* in shrimp caused the total bacterial number to decrease significantly and knockdown of *MjRhoA* increased the bacterial number obviously, with a consequent decline in shrimp survival. These results confirmed the antibacterial function of *Mj*RhoA in shrimp. Further study showed that rate of phagocytosis of hemocytes was decreased in *MjRhoA*-knockdown shrimp. Interestingly, we observed that *Mj*RhoA was translocated onto the hemocyte membrane at 1 h post *V*. *anguillarum* challenge. The expression levels of the β-integrin-mediated phagocytosis markers *ROCK2* and *Arp2/3* declined significantly after knockdown of *MjRhoA*. These results suggested that the antibacterial function of *Mj*RhoA was related to β-integrin-mediated phagocytosis in shrimp. Our previous study identified that a C-type lectin, hFcLec4, initiated β-integrin mediated phagocytosis after bacterial infection. Thus, knockdown of hFcLec4 and β-integrin was performed. The results showed that the translocation of *Mj*RhoA from the cytoplasm to membrane was inhibited and the expression level of *Mj*RhoA was decreased, suggesting that *Mj*RhoA participated in hFcLec4-integrin mediated phagocytosis. Therefore, our study identified a new hFcLec4-integrin-RhoA dependent phagocytosis against bacterial infection in shrimp.

## Introduction

Small GTPases play a crucial role in the biological functions of organisms. To date, many small GTPase members have been identified and all the members belong to the Ras GTPase superfamily (1). Commonly, the Ras superfamily is divided into five groups: Ras, Ran, Rho, Rab, and Arf/Sar families (2). Except for the Arf family, the other four groups exhibit GTPase activity. All members of these families have two states, the active state (GTP-binding state) and the inactive state (GDP-binding state). The two states can be reversed by guanine nucleotide exchange factors (GEF) and GTPase-activating proteins (GAP). Through this transformation between the active and inactive states, the small GTPases successfully regulate their downstream effectors (3).

The Rho GTPase family consists of three major members, Rho, Rac, and Cdc42 (cell division cycle 42). The main functions of Rho GTPases are regulating cytoskeleton regrouping, the cell cycle, gene expression, and signal transduction (4). In particular, Cdc42 mainly functions in cell apoptosis (5), cell polarity (6), and cell motility (7). Cdc42 and Rac also regulate filopodia formation synergistically (8). In addition, various signaling pathways are regulated by Rho GTPases; for example, Cdc42 together with Rac1 regulates the transcriptional activity of the transcription factor c-Jun in JNK signaling pathway activation (9). Particular to animal cells (10), Rac1 plays a crucial role in activating the nuclear factor kappa B (NF-κB) signaling pathway via the Toll-like receptor 2 in its activated state (11). As another key member of Rho GTPases, RhoA functions in regulating the actin cytoskeleton (12). The actin cytoskeleton is crucial for cell movement, material transport, and cell differentiation by regulating the morphological structure of cells (13). In vertebrates, the expression level of RhoA is much higher in tumor cells than in normal cells (14), indicating a close association with tumor progression. In fact, RhoA-dependent cytoskeleton regulation is a fundamental molecular mechanism in tumorigenesis (15).

In contrast to vertebrates, there have been few studies of the functions of Rho GTPases family members in invertebrates. For example, Rac1 has dual functions in antibacterial and antiviral innate immunity in *Fenneropenaeus chinensis* (16); Cdc42 inhibits the replication of the DNA virus white spot syndrome virus (WSSV) by interacting with the arginine kinase in kuruma shrimp *Marsupenaeus japonicus* (17). However, there are no reports about the function of Rho in invertebrates.

In the present study, we identified a member of the Rho GTPases, RhoA, using transcriptome sequencing of kuruma shrimp, *M. japonicus* (denoted hereafter as *Mj*RhoA). We observed that *MjRhoA* was highly expressed in the *Vibrio anguillarum*-challenged shrimp hemocytes and heart. Knockdown of *MjRhoA* led to high bacterial numbers and high mortality of shrimp. The possible mechanism of *Mj*RhoA function in shrimp was analyzed. The results indicated that RhoA might inhibit bacterial infection by enhancing phagocytosis in shrimp.

## Materials and methods

### Gene cloning and bioinformatic analysis

The sequence of *MjRhoA* was obtained by transcriptome sequencing of hemocytes from *M*. *japonicus*. The sequence identity of *MjRhoA* was analyzed by the online BLASTX algorithm (https://www.ncbi.nlm.nih.gov/). The translation and isoelectric point (pI)/molecular weight (Mw) analysis of the nucleotide sequences were performed using the online tool ExPASy (https://www.expasy.org/). GeneDoc and MEGA6, respectively, were used to perform the sequence alignment and to construct the phylogenetic tree of RhoA.

### Animals, immune challenge, and tissue extraction

Healthy kuruma shrimp, *M. japonicus*, (9–12 g) were purchased in a seafood market in Jinan, Shandong, China. The shrimp were initially cultured in a water tank at 25°C for 2 days for acclimatization to the laboratory conditions. Thereafter, for the immune challenge, the shrimp were injected with *V. anguillarum* (10^7^ colony forming units (CFUs) per shrimp) suspended in 50 μL of sterile phosphate-buffered saline (PBS, 140 mM NaCl, 2.7 mM KCl, 10 mM Na_2_HPO_4_, 1.8 mM KH_2_PO_4_, pH = 7.4). Shrimp injected with same amount of sterile PBS were used as controls.

After the shrimp were challenged by the bacteria, different organs (heart, hepatopancreas, gills, stomach, and intestine) were collected and homogenized using manual homogenizers. To collect the hemocytes, the total hemolymph was collected using a 5 mL syringe containing 1 mL anticoagulant (0.45 M NaCl, 10 mM KCl, 10 mM EDTA, and 10 mM HEPES, pH = 7.45) and then centrifuged at 800 × g for 6 min at 4°C to collect the hemocytes. The obtained hemocytes and the other organs (heart, hepatopancreas, gills, stomach, and intestine) were used for total RNA and total protein extraction.

### Total RNA extraction and cDNA synthesis

The total RNA was isolated using TRIpure reagent (Aidlab, Beijing, China). The obtained organs and hemocytes (10 mg) were homogenized in 1 mL of Trizol reagent for RNA extraction. Based on the previous study in our lab, the cDNA was synthesized using 5 μg total RNA with the SMART cDNA synthesis kit (Clontech) following manufacterer's instuctions. The obtained cDNA was used to detect the expression level of different genes using specific primers (Table 1).

**Table 1:** Primers used in this study.

**Primer**	**Sequence (5′-3′)**
SMART F	TACGGCTGCGAGAAGACGACAGAAGGG
Oligoanchor R	GACCACGCGTATCGATGTCGACT16(A/C/G)
EF1αRTF	GGATTGCCACACCGCTCACA
EF1αRTR	CACAGCCACCGTTTGCTTCAT
*Mj*RhoA RTF	CGTGCCCACAGTATTTG
*Mj*RhoA RTR	ACTTCAGGCGTCCATTT
β-integrin RTF	TTGGCAGAAAACGGAGAAT
β-integrin RTR	TTAGGAGCGTGAGGAGGC
hFcLec4 RTF	TCCTCGGCTGGTTCTGGT
hFcLec4 RTR	ACGAAGGGCTTTTTGTGGTAG
ROCK2 RTF	TGTGAGGTGTGTCAGCGG
ROCK2 RTR	CGACGAAGGGACGGTAAG
Arp2/3 RTF	GTCGTCGTCCGTCTCTTCCC
Arp2/3 RTR	CTTTGGTGGTTGGCAGTCG
*Mj*RhoAexF	CGCGGATCCATGGCGGCCATTAGGAAAAA
*Mj*RhoAexR	CCGCTCGAGTTACAAAAGGGTACACTTAG
*Mj*RhoA RNAiF	GCGTAATACGACTCACTATAGGCTAAGACCAAGGAGGGC
*Mj*RhoA RNAiR	GCGTAATACGACTCACTATAGGGGAGGCAGCAAACTACA
β-integrin RNAiF	GCGTAATACGACTCACTATAGGCTGACAGACTCCTCCCCC
β-integrin RNAiR	GCGTAATACGACTCACTATAGGCAGAACTGCCCTTGGTAAC
hFcLec4 RNAiF	GCGTAATACGACTCACTATAGGGACGATGAGCAGAAGGGC
hFcLec4 RNAiR	GCGTAATACGACTCACTATAGGAAGAACAATGCCCGGGTT
GFP RNAiF	GCGTAATACGACTCACTATAGGTGGTCCCAATTCTCGTGGAAC
GFP RNAiR	GCGTAATACGACTCACTATAGGCTTGAAGTTGACCTTGATGCC

### Semiquantitative RT-PCR analysis and real-time RT-PCR (qPCR) analysis assays

To analyze the expression level of the target gene *MjRhoA* at the transcriptional level, a semiquantitative method and a real-time quantitative method were performed. The semiquantitative RT-PCR was used to detect the tissue distribution of *MjRhoA* using a pair of specific primers (*Mj*RhoARTF and *Mj*RhoARTR). The RT-PCR profile was as follows: 94°C for 3 min; 35 cycles of 94°C for 20 s, 56°C for 30 s, and 72°C for 30 s; followed by 72°C for 10 min. The PCR products were analyzed using agarose gel electrophoresis (AGE) with a 1.5% agarose gel. As a control, the expression level elongation factor 1-alpha (EF1α) was determined in the same way, using a pair of primers (EF1α RTF and EF1α RTR).

To analyze the expression profiles of the target genes, qPCR was performed with primers *Mj*RhoARTF and *Mj*RhoARTR. EF1α RTF and EF1α RTR (Table 1) were used as the internal control. The 10 μL qPCR reaction system contained 5 μL 2 × SYBR qPCR mix, 4 μL of primers (0.5 μM), and 1 μL cDNA. The qPCR profile was as follows: 95°C for 10 min; 40 cycles of 95°C for 15 s, 60°C for 50 s, and reading for 2 s at 72°C; the melting curve stage was from 65 to 95°C. The 2^−ΔΔ*CT*^ method was used to analyze the qPCR data and the results were shown as mean ± SD. Student's *t*-test was used to analyze the significant difference between the PCR data of two groups.

### Recombinant expression, purification, and antiserum preparation

To study the function of *Mj*RhoA at the protein level, we first recombinantly expressed *Mj*RhoA in the *Escherichia coli*. First, the cDNA fragment of *MjRhoA* was amplified using primers, *Mj*RhoAexF and *Mj*RhoAexR (Table 1). Then the cDNA fragment of *MjRhoA* and the empty pET30a (+) vector were digested with *Bam*HI and *Xho*I and then purified using the DNA purification kit (Sangon, Shanghai, China). The fragment was ligated into the digested plasmid using T4 DNA ligase (Thermo Fisher). The constructed recombinant plasmid was then transformed into *E. coli* Rosetta cells for the recombinant expression of *Mj*RhoA (induced using 0.5 mM isopropyl β-D-1-thiogalactopyranoside (IPTG). The recombinant *Mj*RhoA was expressed in the supernatant and was purified by affinity chromatography using a Ni-resin (GenScript, Nanjing, China). The purified recombinant protein was dialyzed for 48 h at 4°C in PBS buffer containing 5% glycerinum.

The purified recombinant *Mj*RhoA (r*Mj*RhoA) was used to prepare antiserum in New Zealand white rabbits. Two subcutaneous injections were performed using 500 μg of protein each time. For the first injection, 500 μg of recombinant *Mj*RhoA protein was mixed thoroughly with the same volume of Complete Freund's Adjuvant (SIGMA). After 3 weeks of sensitization, 500 μg of *Mj*RhoA protein mixed with Incomplete Freund's Adjuvant (SIGMA) was used for the second injection. The antiserum was collected and assessed using western blotting.

### DsRNA preparation and RNA interference assay

To study the function of *Mj*RhoA, RNA interference was employed. For the dsRNA preparation, the dsRNA region was analyzed and searched using Primer Premier 5, and the specific primers *Mj*RhoA RNAi F and *Mj*RhoA RNAi R containing a T7 promoter sequence (GCGTAATACGACTCACTATAGG) were synthesized. The primers were used to amplify the dsRNA template. The template was purified by Chloroform extraction. The dsRNA template was then used for the ds*Mj*RhoA synthesis using T7 RNA polymerase. The 50-μL reaction system contained 2.4 μL of the four NTPs (ATP, CTP, GTP, and UTP), 4 μL of T7 RNA polymerase, 2 μL of RNase inhibitor, 1 μL of dsRNA template, 20 μL of T7 RNA polymerase buffer, and 13 μL of RNase-free water. After incubation at 37°C for 6 h, DNase I was added into the mixture and incubation was continued at 37°C for another 1.5 h to digest the DNA template. The dsRNA of *Mj*RhoA was then extracted using Chloroform. The concentration of the extracted *dsMjRhoA* was detected using a micro-spectrophotometer K5500 (K.O., China). The synthesis of the control *dsGFP* was performed with the same way using the primers GFP RNAi F and GFP RNAi R.

For the *in vivo* RNA interference assay, two groups of healthy intermolting shrimp (9 g each) were prepared and each group contained more than 30 individuals. The shrimp in one group were injected with 30 μg of *dsMjRhoA* (diluted in 50 μL RNase-free water) and the shrimp in the control group were injected with the same amount of *dsGFP* (30 μg). After dsRNA injection for 48 h, at least three individuals were randomly chosen to extract the total RNA using the method detailed above for the RNAi efficiency analysis by qPCR. The remaining shrimp were used for survival rate analysis.

### Survival rate analysis

For the survival rate assay, shrimp were divided to two groups (at least 30 individuals in each group) and injected with *dsMjRhoA* or *dsGFP*. The *dsMjRhoA* injection group and the control (*dsGFP* injection) group were simultaneously challenged with the same amount of *V. anguillarum* (10^7^ CFUs/shrimp). Thereafter, the number dead shrimp were monitored every 24 h and the survival rates of the two groups were calculated.

### The *in vivo* bacteria clearance assay

For the bacteria clearance assay, the experimental groups were first injected with 30 μg *dsMjRhoA* (diluted in 50 μL RNase-free water) for *MjRhoA* RNAi or with 10 μg r*Mj*RhoA (diluted in 50 μL PBS) for *Mj*RhoA overexpression. The shrimp were then injected with *V. anguillarum* suspended in 50 μL sterile PBS (10^7^ CFUs/shrimp) 48 h post *dsMjRhoA* injection or 24 h post r*Mj*RhoA injection. Thirty minutes after injection, the hemolymph from at least three individuals in each group was collected using sterile syringes on a clean bench. To detect the total bacterial number *in vivo*, the Petri dishes containing 2216E medium (0.5% tryptone, 0.1% yeast extract, 0.01% FeCl_3_, 1.5% agar, and 2.4% NaCl) were placed on the clean bench beforehand. For each group, 50 μL shrimp of hemolymph was smeared onto the dish. After culturing at room temperature overnight, the single colonies were counted and the number of CFUs was calculated. Each independent experiment was repeated three times. The results are shown as the mean ± SD and were statistically analyzed using Student's *t*-test. Shrimp injected with *dsGFP* for RNAi and injected with a His Tag for the overexpression assay were used as controls, respectively.

### Immunocytochemistry and phagocytosis assay

For the immunocytochemistry assay, hemocytes were washed with PBS twice and then dropped on a prepared glass slide (coated with polylysine) for 1 h to sediment the hemocytes. The glass slides were then treated with 0.2% Triton X-100 (in PBS) for 5 min to enhance the permeability of the cell membrane. Afterwards, the glass slides were washed with PBS for 5 min six times. The hemocytes were then blocked in 3% bovine serum albumin (BSA) at 37°C for 30 min. The hemocytes were incubated with *Mj*RhoA antiserum as the primary antibody diluted with 3% BSA (1:500) at 37°C for 3 h. Thereafter, the glass slides were washed with PBS for 5 min six times. After blocking in 3% BSA for 30 min for a second time, the hemocytes were incubated with the secondary antibody (fluorescein isothiocyanate (FITC)-goat anti rabbit, diluted with 3% BSA, 1:10,000) at 37°C for 1 h. The slides were then washed with PBS six times, and the cell nuclei were stained with 2-(4-amidinophenyl)-1H-indole-6-carboxamidine (DAPI) (diluted with PBS, 1:1,000) for 10 min. After washing with PBS six times, the hemocytes were observed under a fluorescence microscope (Olympus BX51, Japan).

For the phagocytosis assay, untreated shrimp were first injected with FITC-labeled *V. anguillarum*. To prepare the FITC-labeled *V. anguillarum*, the overnight cultured bacteria were first killed at 72°C for 20 min, and then suspended in 0.1 M NaHCO_3_ (pH = 9.0); the bacteria were labeled with 1 mg/mL FITC at 28°C for 1 h. After washing with sterile PBS six times (5 min each), the bacteria were injected into shrimp. Thirty minutes after injection, the shrimp hemocytes from different groups were collected and washed with PBS twice. The hemocytes were dropped onto a glass slide (coated with polylysine) for 1 h. After treatment with 0.2% Triton X-100 (in PBS) for 5 min, the cells were washed with PBS five times. The nuclei was stained with DAPI for 10 min. After washing with PBS, the hemocytes were observed under a fluorescence microscope and the phagocytosis rate (the proportion of phagocytic cells among the total cells) and the phagocytic index (the proportion of bacteria phagocytosed by the shrimp hemocytes among the total cells) were calculated.

## Results

### Sequence alignment and phylogenetic tree of RhoA

The mRNA sequence of *RhoA* was obtained by transcriptome sequencing in kuruma shrimp *M. japonicus* and was named *MjRhoA*. The full-length cDNA sequence of *MjRhoA* consists of 1,640 bp, with a 579 bp open reading frame (Figure S1). *MjRhoA* encodes a polypeptide of 192 amino acids, with theoretical pI and Mw of polypeptide 6.0 and 21.60 KDa.

To analyze the sequence similarity with RhoAs from different species, sequence alignment was performed and a phylogenetic tree was built. The analysis was showed that RhoAs from different species are relatively conserved (Figure S2A). Notably, Rho1 proteins from *D. melanogaster* and other species were quite similar to RhoA, suggesting that Rho1s belonging to the RhoA family. In the phylogenetic analysis, the RhoAs proteins were mainly divided into two branches: RhoAs of invertebrates and RhoAs of most vertebrates. Though *Mj*RhoA is phylogenetically close to those RhoAs of different invertebrates, it belongs to one specific branch (Figure S2B). It is worth mentioning that the RhoA from the freshwater fish *Poeciliopsis prolifica* is quite different from the other RhoAs (Figure S2B).

### *Mj*RhoA was upregulated in shrimp challenged by *V. anguillarum*

**Figure 1:**
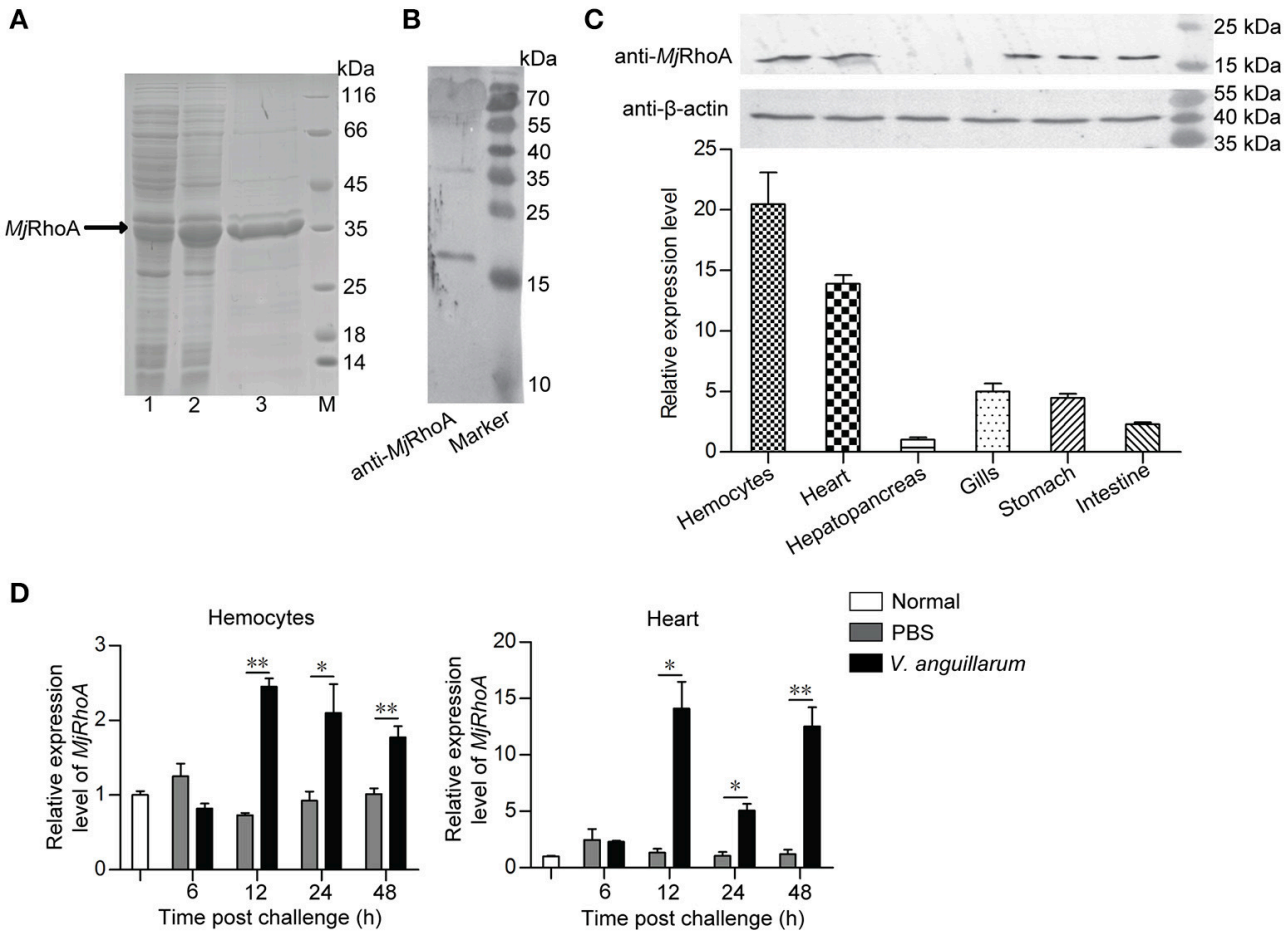
*Mj*RhoA was upregulated in shrimp challenged by *V. anguillarum*. **(A)** Recombinant expression of *Mj*RhoA in *E. coli* cells detected by SDS-PAGE. Lane 1, total proteins in the *E. coli* Rosetta cells transformed with pET30a-*MjRhoA* before induction with IPTG; Lane 2, total proteins in the *E. coli* Rosetta cells containing pET30a-*MjRhoA* after induction with 0.5 mM IPTG; Lane 3, purified recombinant *Mj*RhoA using affinity chromatography with a His-binding resin; Lane M, protein standard. **(B)** The specificity of the anti-*Mj*RhoA prepared using the recombinant *Mj*RhoA was detected by western blotting. **(C)** Expression level of *Mj*RhoA in different tissues of normal shrimp. The expression level of *Mj*RhoA in hemocytes and other five tissues (heart, hepatopancreas, gills, stomach, and intestine) were detected by western blot (upper) and qPCR (bottom). In the qPCR results, the relative expression levels in different tissues were normalized by the lowest expression level of *MjRhoA* in the hepatopancreas. **(D)** The expression patterns of *MjRhoA* at different time points post *V. anguillarum* challenge. Untreated shrimp and the shrimp injected with PBS were used as controls. All the results were from at least three repeated experiments and are shown as the mean ± SD. The significance of the differences was analyzed using Student's *t*-test. *p < 0.05, **p < 0.01.

First, polyclonal antibodies against *Mj*RhoA was prepared using r*Mj*RhoA with a His tag (Figure 1A). The anti-*Mj*RhoA polyclonal antibodies could recognize native *Mj*RhoA in shrimp with appropriate molecular mass although there were also a few non-specific bands recognized by the antibodies (Figure 1B), this might be the impurity of the protein. The tissue distribution of *Mj*RhoA in shrimp was then analyzed by qPCR and western blotting. The results showed that *MjRhoA* was expressed in different tested tissues and was highly expressed in hemocytes and heart, with quite low expression in the hepatopancreas (Figure 1C). The expression patterns of *MjRhoA* in hemocytes and heart were detected using qPCR. The results indicated that the expression of *MjRhoA* was upregulated in hemocytes at 12 h post *V. anguillarum* challenge and remained at a high level from 12 to 48 h (Figure 1D). The expression level of *MjRhoA* was also upregulated in the heart at 12 h post *V. anguillarum* infection, and the expression level at 24 h post infection was relatively lower than 12 h and 48 h; however, it was still higher than that in the normal (unchallenged) control samples (Figure 1D). These results indicated that *MjRhoA* might play a role in resisting infection by *V. anguillarum* in shrimp.

### *Mj*RhoA suppresses bacterial infection *in vivo*

**Figure 2:**
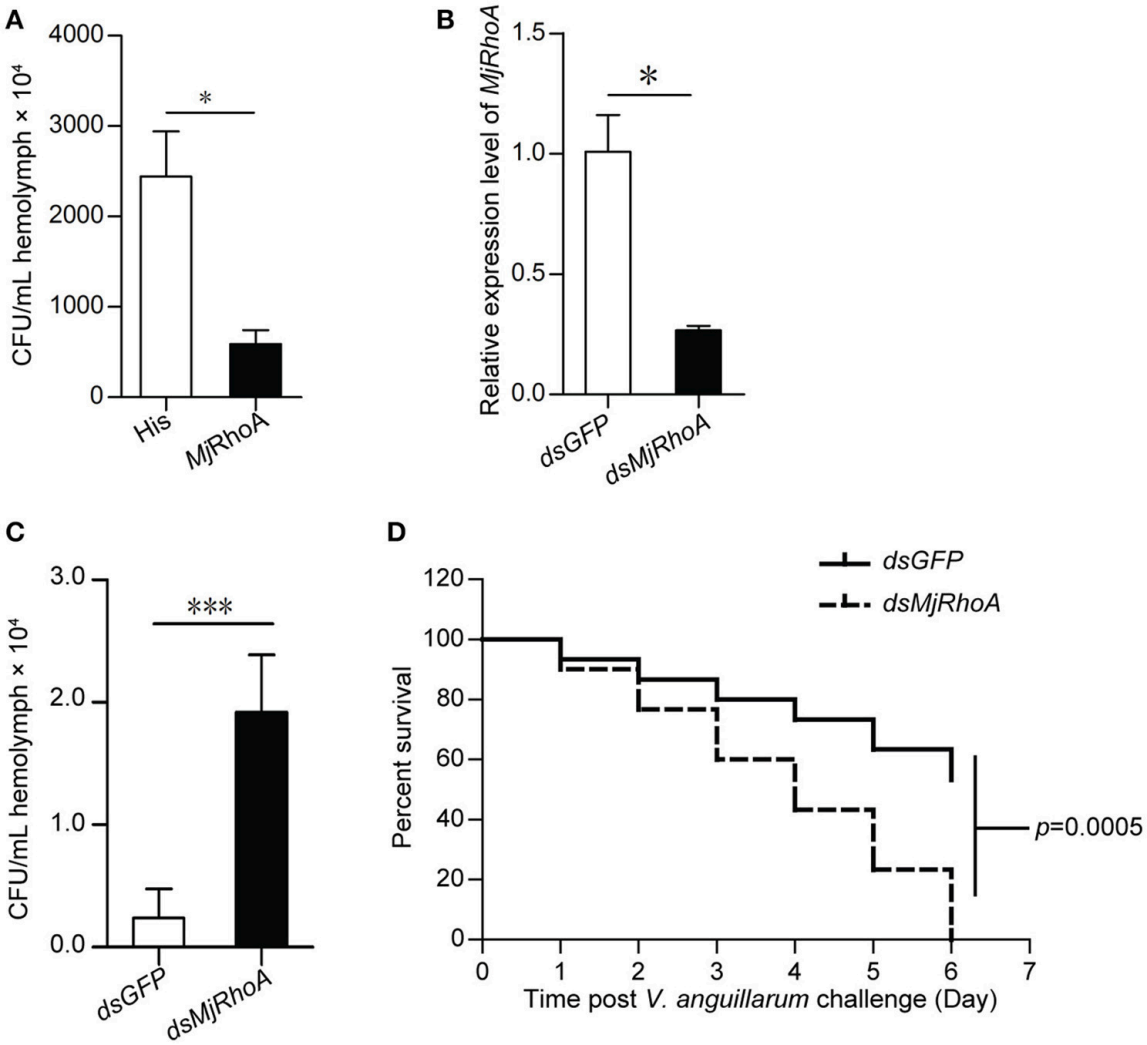
The total bacteria number and survival rate analysis after overexpression and knockdown of *Mj*RhoA. **(A)** Total bacteria detected after treatment with *Mj*RhoA (“overexpression”) in the shrimp infected with *V. anguillarum*. After overexpression of *Mj*RhoA, the total bacteria from the whole hemolymph were counted. The bacteria number in the His-tag injection group was used as a control. **(B)** The efficiency of *MjRhoA* RNAi. Shrimp were injected with 30 μg of *dsMjRhoA* and the same amount of *dsGFP*, respectively. After 48 h, the shrimp hemocytes were collected and the total RNA was extracted for qPCR analysis. Shrimp injected with *dsGFP* were used as a control. **(C)** Total bacteria *in vivo* detecting after knockdown of *MjRhoA*. The whole hemolymph of shrimp were collected and spread onto the LB solid medium. After growing at room temperature, the total bacteria were counted. The total bacteria number in the *dsGFP* injected shrimp was used as a control. **(D)** Survival rate of shrimp infected with *V. anguillarum* after knockdown of *MjRhoA*. The survival rate of shrimp injected with *dsGFP* was used as a control. The data were statistically analyzed using Student's *t*-test. *p < 0.05, ***p < 0.001.

To investigate the function of *Mj*RhoA in bacterial infection, overexpression and knockdown of *MjRhoA* were performed, and the bacterial number in the shrimp was analyzed. For the overexpression, we injected 10 μg r*Mj*RhoA and the His tag into two shrimp groups, separately, before *V. anguillarum* infection and then the total bacterial number was analyzed. After overexpression of *Mj*RhoA, the number of bacteria *in vivo* was significantly decreased compared with that in the control (Figure 2A). To confirm the function of *Mj*RhoA, we then performed an RNAi assay. After knockdown of *MjRhoA* (Figure 2B), we assessed the bacterial number in the hemolymph of the shrimp. The result showed the number of total bacteria *in vivo* was significantly increased after knockdown of *MjRhoA* (Figure 2C) compared with that in the control. To analyze the survival rate, shrimp were divided into two groups (shrimp injected with *dsMjRhoA* as the experimental group and shrimp injected with *dsGFP* as the control), followed by infection with *V. anguillarum*. As shown in Figure 2D, after knockdown of *MjRhoA* expression, the survival rate was markedly decreased compared with that in the *dsGFP* group (Figure 2D). These results indicated that the small GTPase *Mj*RhoA contributes to resistance to bacterial infection *in vivo*.

### The bacterial clearance ability of *Mj*RhoA was related to phagocytosis

**Figure 3:**
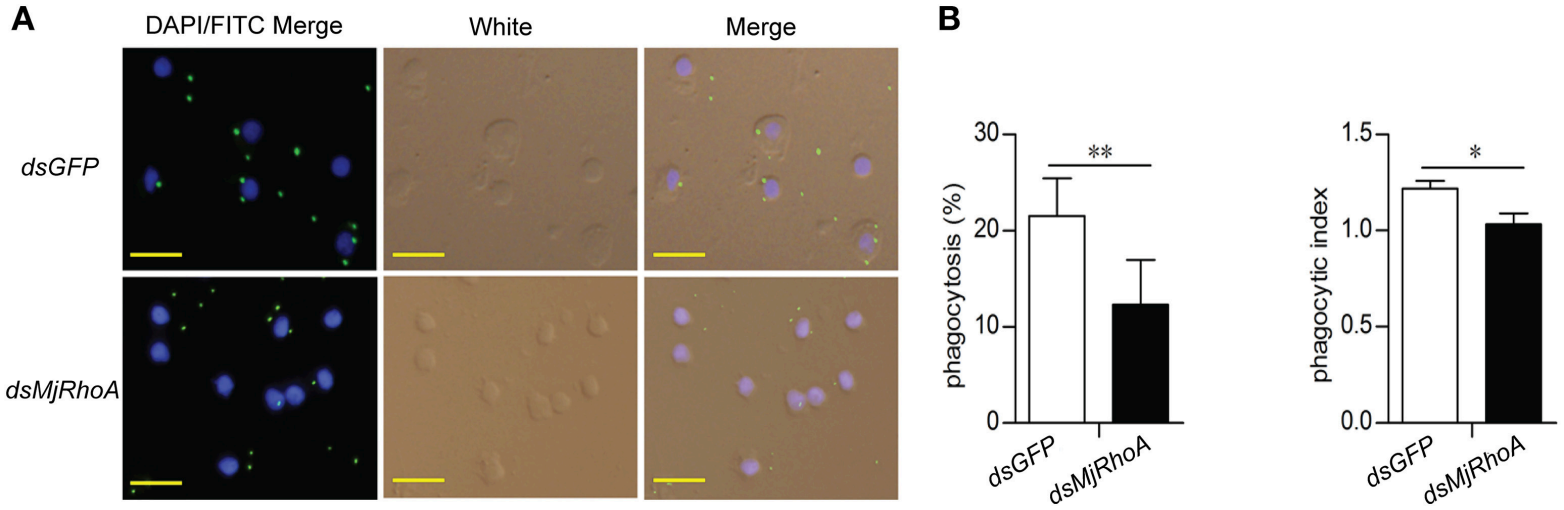
*Mj*RhoA bacterial clearance was dependent on phagocytosis. **(A)** Bacterial phagocytosis of hemocytes was analyzed by immunocytochemistry. The phagocytosis detection with hemocytes from *dsGFP*-injected shrimp was used as a control. **(B)** The phagocytosis rate and the phagocytic index calculated after counting hemocytes injected with FITC- *V. anguillarum*. At least 100 cells were counted and three different views were chosen for statistical analysis. *p < 0.05, **p < 0.01.

To analyze the cellular mechanism of *Mj*RhoA's bacterial clearance function, we performed immunocytochemistry in hemocytes from *MjRhoA*-knockdown shrimp injected with FITC labeled *V. anguillarum*. The results showed that the amount of the FITC-labeled bacteria in the hemocytes was reduced in *MjRhoA*-knockdown shrimp compared with that in the control (Figure 3A). The phagocytosis rate and phagocytic index were then analyzed. The results showed that both of the phagocytosis rate and the phagocytic index decreased after knockdown of *MjRhoA* (Figure 3B). These results suggested that the bacterial clearance ability of *Mj*RhoA was dependent on hemocyte phagocytosis.

### *V. anguillarum* challenge affects the subcellular location of *Mj*RhoA in hemocytes

**Figure 4:**
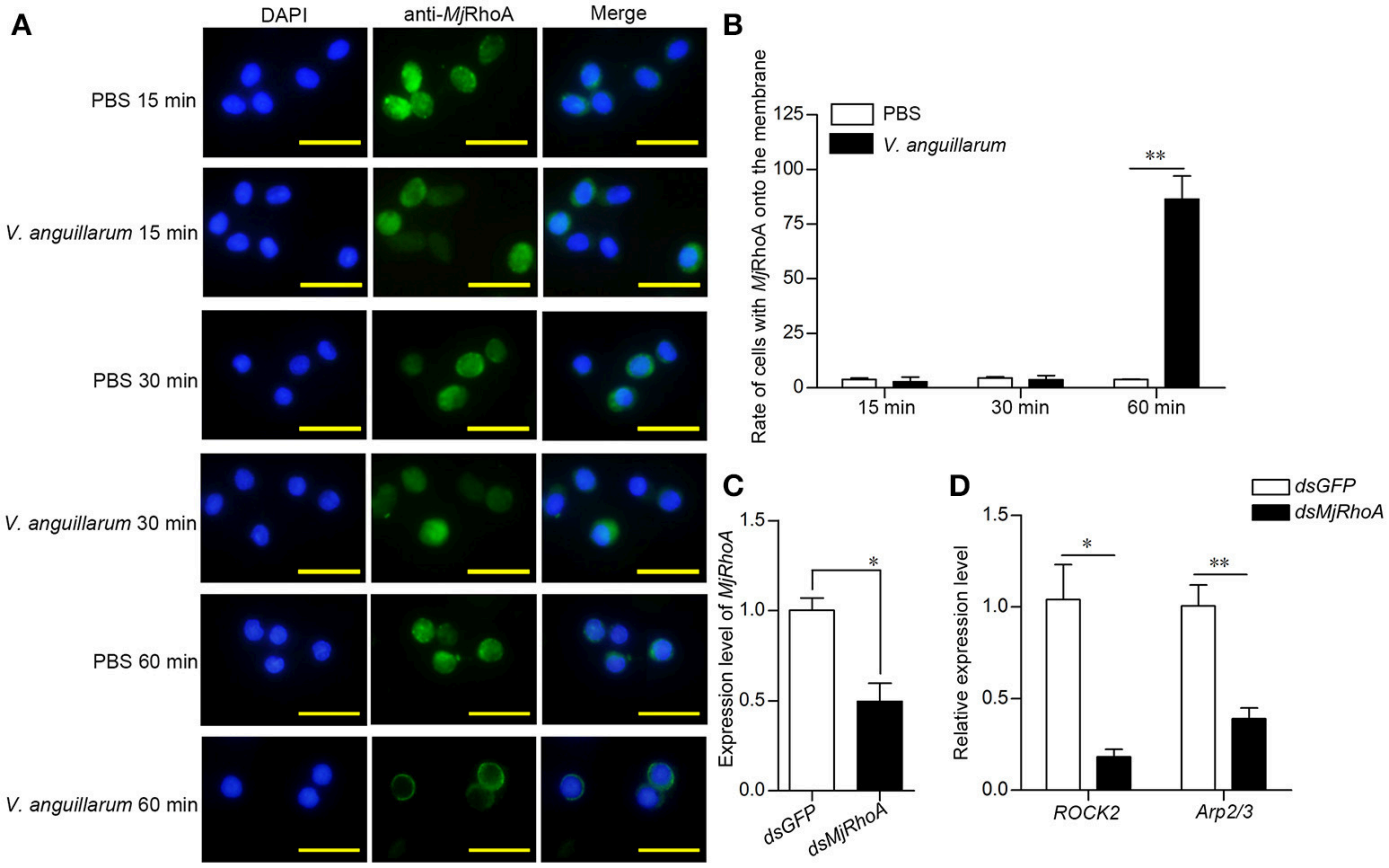
*Mj*RhoA transfers to the cell membrane and regulates phagocytosis related genes after bacterial challenge. **(A)** The location of native *Mj*RhoA in hemocytes, detected using immunocytochemistry. Normal shrimp were injected with 50 μL *V. anguillarum* (10^7^ cells per shrimp) and the same volume of PBS for 1 h, separately. The *Mj*RhoA location *in vivo* was detected using anti-*Mj*RhoA antiserum. The nuclei were stained with DAPI. The location of *Mj*RhoA in PBS-injected shrimp hemocytes was used as a control. Scale bar = 20 μm. **(B)** The proportion of hemocytes with *Mj*RhoA on the cell membrane among the total cells among shrimp challenged by *V. anguillarum* at different time points. Shrimp injected with PBS was used as a control. **(C)** The RNAi efficiency of *MjRhoA* after knockdown for 48 h. Shrimp injected with *dsGFP* were used as a control. **(D)** The expression level of phagocytosis-related genes was detected using qPCR after knockdown of *MjRhoA*. Two genes, *ROCK2* and *Arp2/3*, were chosen to detect phagocytosis. *DsGFP* injection was injected as a control. *p < 0.05, **p < 0.01.

Although the above results indicated that the anti-bacterial function of *Mj*RhoA was related to phagocytosis, the molecular mechanism was still unclear. Rho GTPases family members are recruited to the phagocytic site and converted from their inactive GDP-bound forms to their active GTP-bound forms in the process of phagocytosis (18). We first analyzed the subcellular distribution of *Mj*RhoA in shrimp after bacterial challenge. We found that *Mj*RhoA was located in the cytoplasm under normal conditions, and showed no obvious change in hemocytes at the early stage of *V. anguillarum* infection (Figures 4A,B). *Mj*RhoA translocated onto the membrane of the hemocytes at 1 h post *V. anguillarum* challenge, whereas it was still in the cytoplasm in the PBS-treated group (Figures 4A,B). We then analyzed the expression level of genes related to phagocytosis (*ROCK2* and *Arp2/3*). The expression levels of these phagocytosis-related genes were downregulated (Figure 4D) after knockdown of *MjRhoA* (Figure 4C). The results confirmed that *Mj*RhoA responds to bacterial infection and exerts its anti-bacterial function via phagocytosis.

### *Mj*RhoA is required for β-integrin-mediated phagocytosis

**Figure 5:**
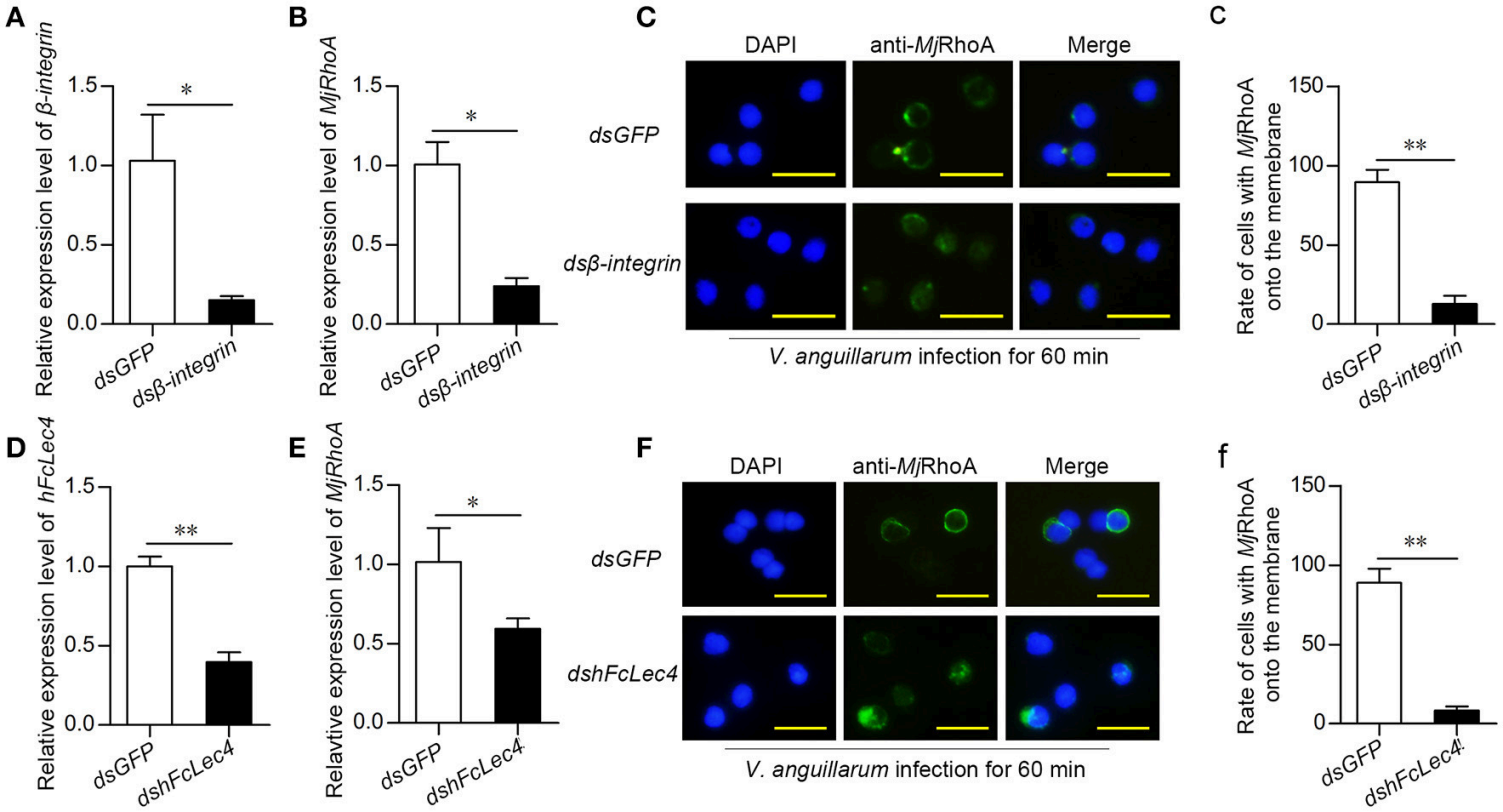
*Mj*RhoA participates in hFcLec4-β-integrin-mediated phagocytosis. **(A)** The knockdown efficiency after injection of β*-integrin* dsRNA. Shrimp injected with the same amount of *dsGFP* (30 μg per shrimp) were used as a control. **(B)** *MjRhoA* expression detection after knockdown of β*-integrin*; *dsGFP* was used as a control. **(C)** The subcellular location of *Mj*RhoA analysis by immunocytochemistry assay after knockdown of *ds*β*-integrin*. Normal shrimp were first injected with 30 μg β*-integrin* dsRNA for 48 h and then injected with *V. anguillarum* (10^7^ cells per shrimp) for 1 h. The location of *Mj*RhoA in *dsGFP*-injected shrimp hemocytes was used as a control. Scale bar = 20 μm. **(c)** The statistical analysis of the cells with *Mj*RhoA located on the membrane after injection of *ds*β*-integrin*; at least three different views were chosen for cell counting; *dsGFP* injection was used as a control. **(D)** The knockdown efficiency of *hFcLec4*. Injection of *dsGFP* was used as a control. **(E)** The *MjRhoA* expression level analyzed by qPCR after knockdown of *hFcLec4; dsGFP* was used as a control. **(F)** The subcellular location of *Mj*RhoA after knockdown of *hFcLec4*; *dsGFP* was used as a control. **(f)** Rate of cells with *Mj*RhoA transferred to the cell membrane after knockdown of *hFcLec4*. *p < 0.05, **p < 0.01.

ROCK2 and Arp2/3 were reported as two crucial members of the integrin-mediated phagocytosis pathway (19, 20). In addition, RhoA is related to integrin-mediated phagocytosis (21, 22). Thus, we detected the expression level and subcellular location of *Mj*RhoA after knockdown of β*-integrin* followed by *V. anguillarum* infection for 60 min. After knockdown of β*-integrin* (Figure 5A), *Mj*RhoA expression was also decreased (Figure 5B), indicating that β*-integrin* could regulate the downstream *Mj*RhoA. *Mj*RhoA was located on the cell membrane in the *dsGFP*-injection shrimp 1 h post *V. anguillarum* challenge (Figures 5C,c). However, the translocation of *Mj*RhoA to the cell membrane in hemocytes was inhibited after knockdown of β*-integrin* in shrimp challenged by the bacteria (Figure 5C). These results suggested that *Mj*RhoA participates in β-integrin-dependent phagocytosis of hemocytes against *V*. *anguillarum* infection.

### FcLec4 homolog is the pattern recognition receptor in β-integrin-RhoA-mediated phagocytosis

A previous study showed that the C-type lectin, hFcLec4, interacts with β-integrin to promote hemocyte phagocytosis (23). To confirm that *Mj*RhoA participates in hFcLec4-integrin-mediated phagocytosis, we detected the effects of hFcLec4 on the expression level and subcellular location of *Mj*RhoA. The results showed that *MjRhoA* expression was downregulated after knockdown of hFcLec4 (Figures 5D,E). As expected, the transfer of *Mj*RhoA onto the membrane was also suppressed after knockdown of hFcLec4 (Figures 5F,f). Taken together, our results identified a new hFcLec4-β-integrin-RhoA-mediated phagocytic pathway against bacterial infection. The intracellular hFcLec4 works as a pattern recognition receptor to sense bacterial infection. After binding to infecting bacteria, hFcLec4 interacts with β-integrin and initiates *Mj*RhoA-mediated phagocytosis.

## Discussion

The present study found that a member of the Rho GTPases, *Mj*RhoA, participates in hFcLec4-β-integrin-mediated hemocyte phagocytosis to protect the host from infection by *V. anguillarum*. β-Integrin has been identified to play a role in anti-microbial immunity in *Drosophila* (24). However, the *Drosophila* integrin that is involved in bacterial uptake is still unknown. An ortholog of the integrin (BINT2) that has been identified in the mosquito *Anopheles gambia* is related to phagocytosis because its gene knockout resulted in reduced phagocytosis of *E. coli* by 70% (25). In the previous study, we identified a C-type Lectin, hFcLec4, which interacts with β-integrin and initiates phagocytosis against bacteria (23). We hypothesized that *Mj*RhoA might be involved in hFcLec4-integrin-mediated phagocytosis. Therefore, we performed RNAi to knock down hFcLec4 and β-integrin expression, and found that the expression level of *Mj*RhoA decreased and subcellular location change of *Mj*RhoA in response to bacterial challenge was inhibited. These results indicated that *Mj*RhoA was involved in the phagocytotic pathway and located in the downstream section.

RNA interference is now a common method of choice for researchers doing for loss of function studies for its ease, speed, and specificity. It is worth mentioning that the knockdown efficiency is sometimes related to different genes: some genes are easily knockdown at low dosage of dsRNA, but some other genes are not even using high dosage of dsRNA. Thus, the injection dosage of dsRNA depends on different situations. For example, 120 μg of *dsPl-TEP* was injected into crayfish (20 ± 2 g) for the RNAi of Pl-TEP (26); in *Penaeus monodon*, the dosage of *PmTBC1D20* dsRNA was 10 μg/g shrimp used in the RNAi assay of *PmTBC1D20* (27). Here in our study, we injected 30 μg dsRNA/shrimp to get the optimal RNAi efficiency.

There are many reports in vertebrates about RhoA functions in cell morphology. In multiciliated epithelial cells (MCCs), RhoA regulates actin assembly and functions in the apical emergence of MCCs (28). In endothelial cells, the activation of local RhoA regulates the F-actin structure formation (29). In addition, there is a kind of synergistic effect between RhoA and actomyosin in regulating the cell-cell junction and functions (30). In vertebrates, the major functions of RhoA are regulating the actin cytoskeleton (31), stress fiber formation (32), and cell polarization (33). In the present study, we found that knockdown of *MjRhoA* inhibited the phagocytosis rate of shrimp hemocytes after injection of *V. anguillarum*. At the same time, the expression levels of phagocytosis-related genes (*ROCK2* and *Arp2/3*) were also downregulated. These results indicated that *Mj*RhoA suppresses bacterial infection in a phagocytosis-related manner. Coincidently, many small GTPases participate in innate immunity in the same manner. Multiple members of the Rab family function in the phagocytosis (34); Rab6 rearranges actin to regulate phagocytosis and protect kuruma shrimp *M. japonicus* from WSSV infection in Ye et al. (35). As a feedback regulation gene (36), Ran interacts with myosin and regulates hemocytic phagocytosis (37). Ran GTPases function as a kind of immunostimulant target that affects phagocytosis against WSSV infection (38). However, all above studies have not referred to the initial step of the phagocytosis, how infected pathogens are recognized? In this study, we provided data for connection of pathogen recognition and phagocytosis in immune response against bacterial infection.

A previous study reported two types of phagocytosis regulated by Rho GTPases (39): Fc gamma receptor (Fcγ)-mediated phagocytosis and integrin-mediated phagocytosis. The first type is regulated by Cdc42 and Rac (40), and another small GTPase, ARF6, is necessary for this type of phagocytosis (41). For integrin-mediated phagocytosis, RhoA plays a crucial role in the pathway and functions in actin polymerization (42). Electron microscopy observation showed that integrin-mediated phagocytosis is not associated with the formation of membrane protrusions (22). Phagocytosis is regulated by RhoA activity, in which activated RhoA is recruited to the site of phagocytosis, and particles that are bound to integrin sink into the cells without generating major protrusions (21). Our results showed that *Mj*RhoA was transferred onto the cytomembrane after *V. anguillarum* challenge, suggesting that a GTP-bound active form of *Mj*RhoA should take part in this type of phagocytosis. After being activated by the upstream integrin, RhoA regulates actin remodeling and then initiates phagosome formation via the downstream effectors, ROCK and Arp2/3 (22). ROCK activity mediates the local recruitment of the actin-branching protein Arp2/3 and the activation of myosin IIA underneath the bound particles (43). As two markers of the β-integrin-mediated phagocytosis pathway (19, 20, 44), the expression levels of *ROCK2* and *Arp2/3* were affected by changes in *Mj*RhoA levels in the present study (Figure 4D).

**Figure 6:**
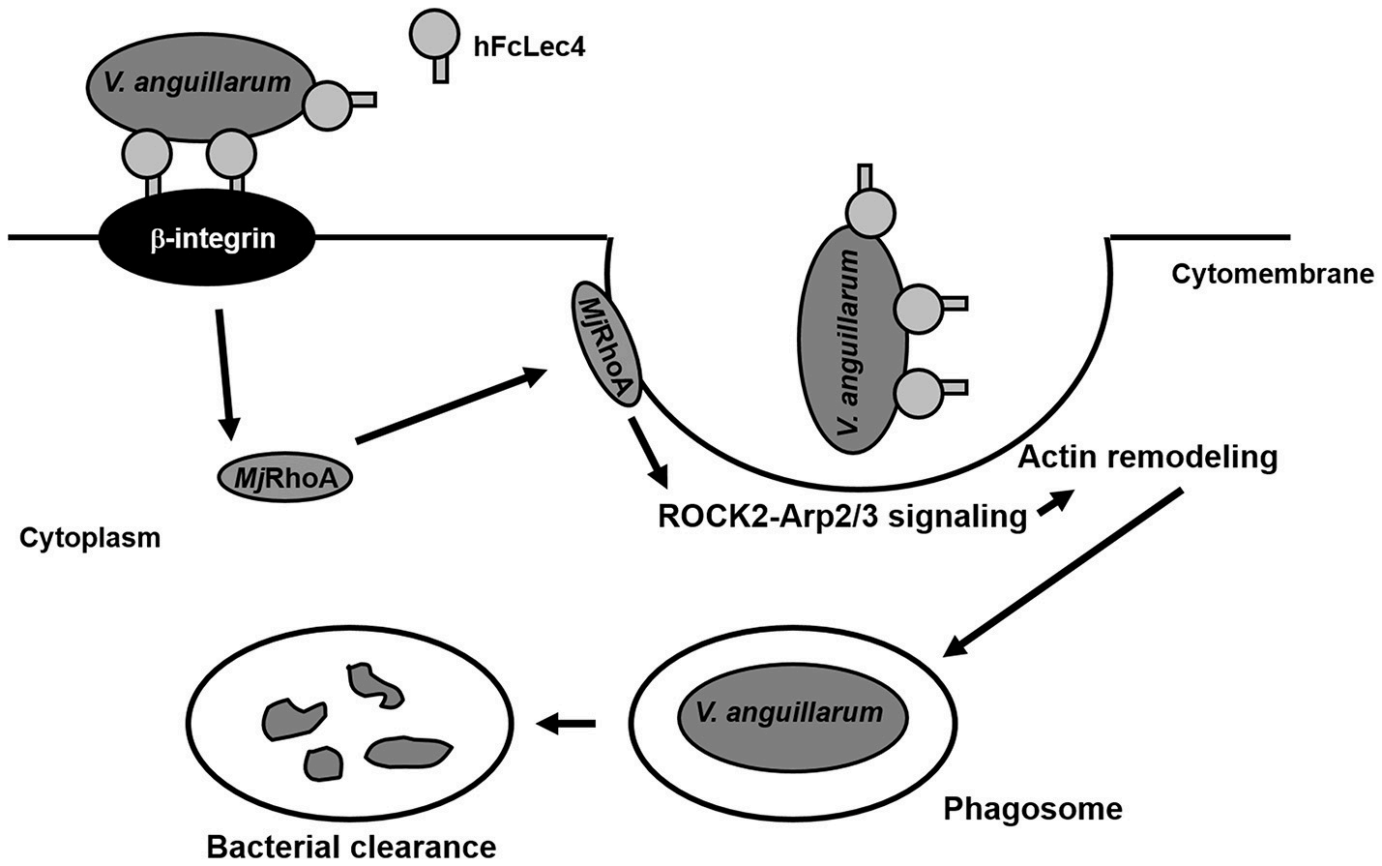
*Mj*RhoA participates in hFcLec4-β-integrin-mediated phagocytosis in shrimp hemocytes during bacterial infection. FcLec4 homolog serves as a pattern recognition receptor (PRR) and recognizes the infecting bacteria. After binding to the bacterial surface, hFcLec4 activates β-integrin signaling and regulates the expression and subcellular location of downstream *Mj*RhoA. Activated *Mj*RhoA is recruited onto the cytomembrane and regulates actin remodeling through the downstream effectors, ROCK2 and Arp2/3. Actin remodeling triggers cell morphological changes and phagosome formation to destroy the bacteria.

In summary, our study found that RhoA participates in hFcLec4-β-integrin-mediated phagocytosis, which protects the host against bacterial infection (Figure 6). Our study identified a new hFcLec4-β-integrin-RhoA mediated phagocytic pathway against bacterial infection in shrimp innate immunity.

## Author contributions

J-XW, X-FZ, and J-DX conceived and designed the experiments. J-DX and J-XW wrote the manuscript. J-DX performed the majority of the experiments. J-DX, J-XW, X-FZ, X-WW, G-JN, and M-QD contributed experimental suggestions and revised the manuscript.

### Conflict of interest statement

The authors declare that the research was conducted in the absence of any commercial or financial relationships that could be construed as a potential conflict of interest.

## Supplementary material

Figure S1 The full-length nucleotide sequence of *MjRhoA* cDNA and predicted amino acid sequence of *Mj*RhoA.

Figure S2 Multiple sequence alignment and phylogenetic tree of *MjRhoA*. **(A)** Multiple sequence alignment of *MjRhoA* with *RhoAs* of 19 other species: *Acanthaster planci, Callorhinchus milii, Ciona intestinalis, Danaus plexippus, Danio rerio, Drosophila melanogaste, Felis catus, Gallus gallus, Homo sapiens, Meleagris gallopavo, Mus musculus, Myotis lucifugus, Ovis aries, Papilio xuthus, Poeciliopsis prolifica, Strongylocentrotus intermedius, Sus scrofa, Tigriopus japonicus, Xenopus laevis*. **(B)** Phylogenetic tree of *MjRhoA* with *RhoA* amino acid sequences of 19 other species.
