# Supplementary figures and images for: A Small GTPase, RhoA, Inhibits Bacterial Infection Through Integrin Mediated Phagocytosis in Invertebrates
Source: Front Immunol. 2018 Aug 30;9:1928. doi: 10.3389/fimmu.2018.01928 (PMC6127615; doi:10.3389/fimmu.2018.01928)

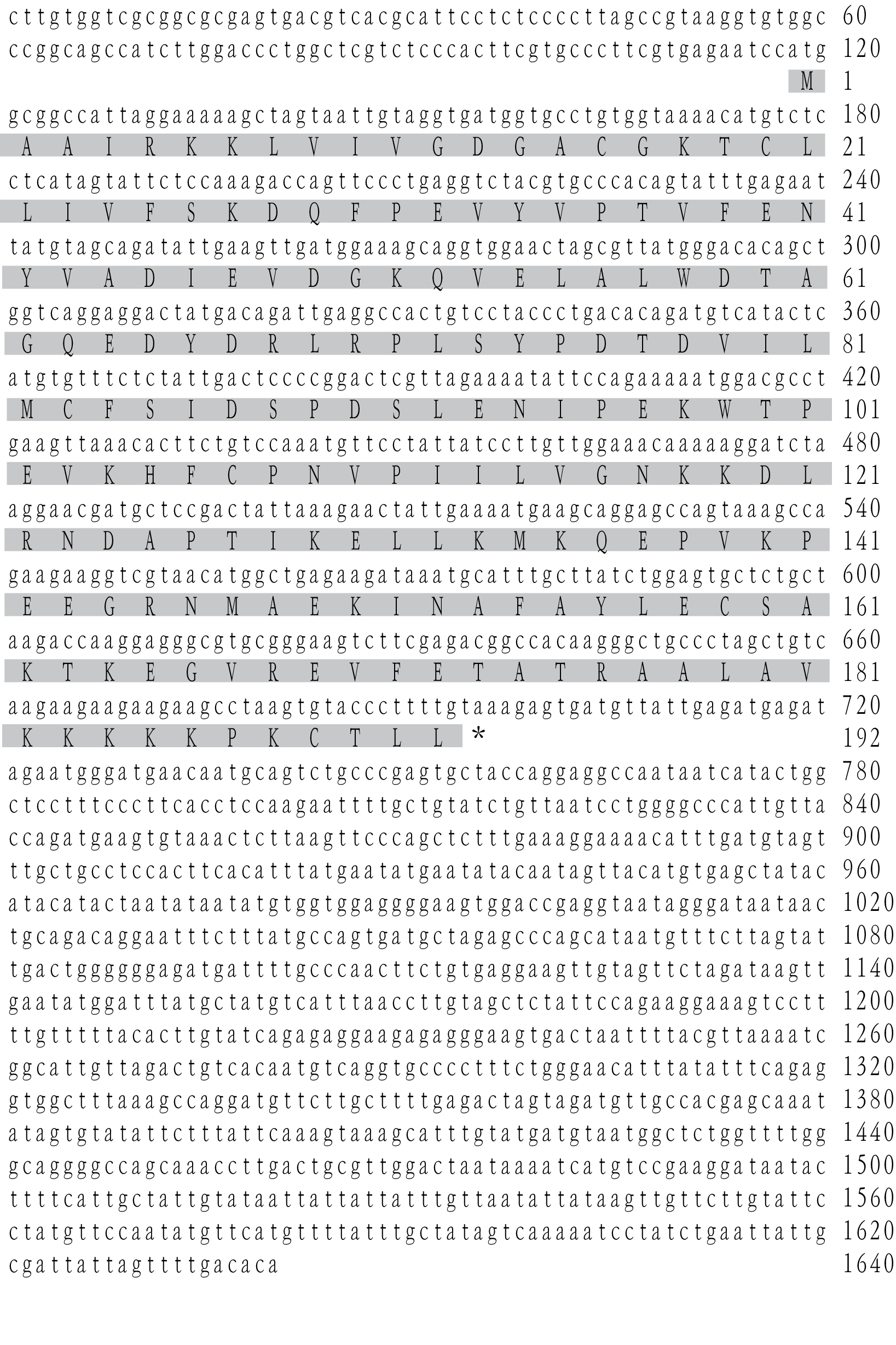

Supplement: Figure S1 — The full-length nucleotide sequence of MjRhoA cDNA and predicted amino acid sequence of MjRhoA. [file Image_1.TIF]

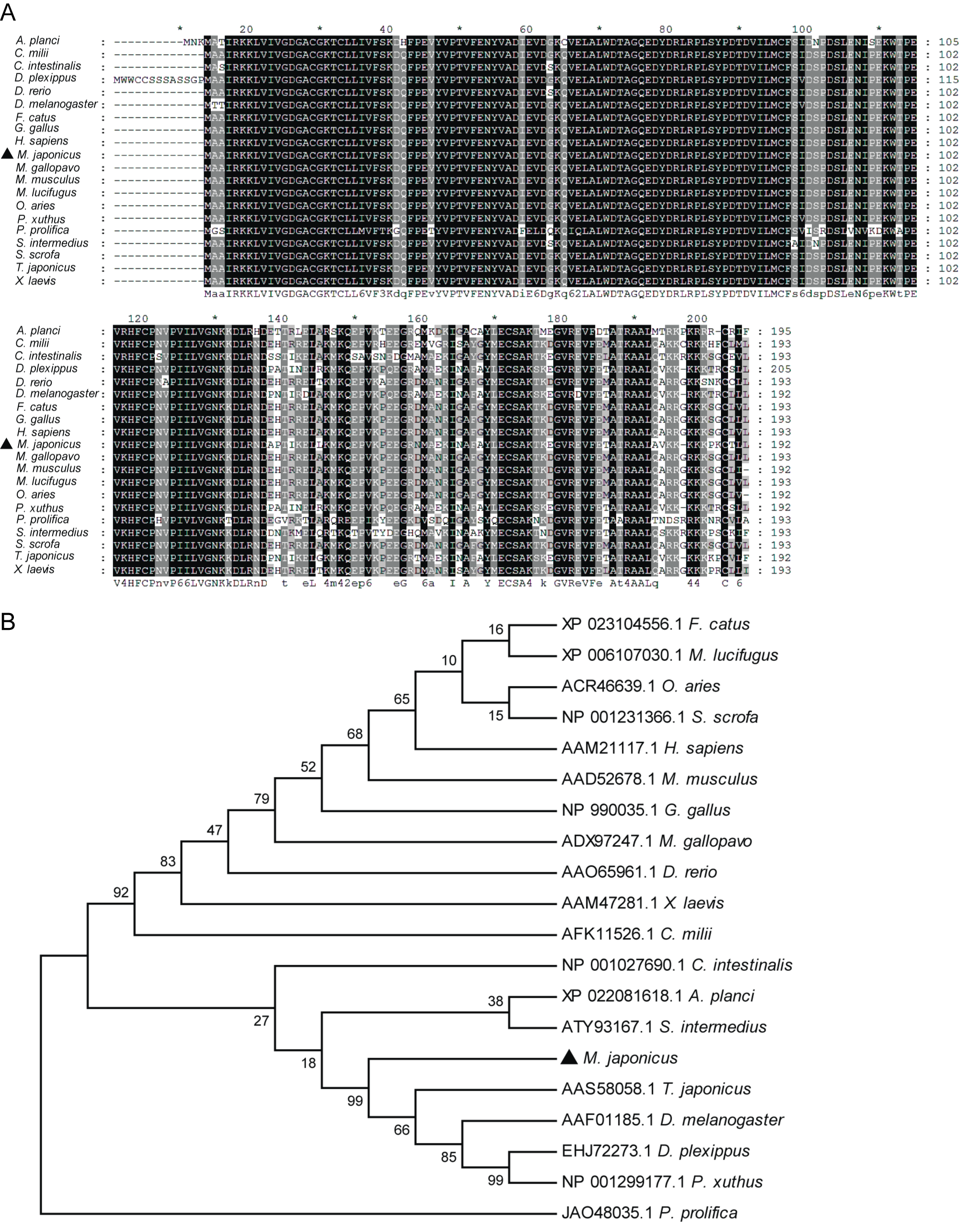

Supplement: Figure S2 — Multiple sequence alignment and phylogenetic tree of MjRhoA. (A) Multiple sequence alignment of MjRhoA with RhoAs of 19 other species: Acanthaster planci, Callorhinchus milii, Ciona intestinalis, Danaus plexippus, Danio rerio, Drosophila melanogaste, Felis catus, Gallus gallus, Homo sapiens, Meleagris gallopavo, Mus musculus, Myotis lucifugus, Ovis aries, Papilio xuthus, Poeciliopsis prolifica, Strongylocentrotus intermedius, Sus scrofa, Tigriopus japonicus, Xenopus laevis. (B) Phylogenetic tree of MjRhoA with RhoA amino acid sequences of 19 other species. [file Image_2.TIF]
